# Real-world outcomes following dehydrated human amnion/chorion membrane use in left-sided colectomy without a protective stoma: a multicenter pilot study

**DOI:** 10.3389/fsurg.2026.1792507

**Published:** 2026-09-09

**Authors:** Bidhan B. Das, Nezar Jrebi, Dennis E. Choat, Taher Sapatwalla, Matthew C. Bardin

**Affiliations:** 1 Southwest Surgical Associates, Houston, TX, United States; 2 North Texas Surgical Specialists, Ft. Worth, TX, United States; 3 Piedmont Physicians Colon and Rectal Surgery of Fayetteville, Fayetteville, GA, United States; 4 Henry Ford Warren Hospital, Warren, MI, United States; 5 STC Clinical, LLC, Tampa, FL, United States; 6 University of Florida College of Pharmacy, Gainesville, FL, United States

**Keywords:** amnion, anastomosis, anastomotic leak, cancer, colectomy, diverticulitis, low anterior resection, sigmoid

## Abstract

**Background:**

Anastomotic leak is a critical complication following colorectal surgery, contributing to reintervention, readmission, prolonged hospitalization, and substantial downstream healthcare costs. Despite advances in surgical technique and perioperative care, leak rates following left-sided colectomy have not meaningfully declined. Dehydrated human amnion/chorion membrane (DHACM) is a placental allograft that contains and releases a broad array of biological mediators implicated in angiogenesis, fibroblast recruitment, and extracellular matrix remodeling. While these properties suggest a potential role in anastomotic healing, clinical data in colorectal surgery remain limited.

**Methods:**

This retrospective, multicenter, observational cohort study evaluated consecutive adult patients who received DHACM during left-sided colectomy with primary colorectal anastomosis and circumferential application of DHACM between January 2016 and June 2024. Patients receiving a protective stoma were excluded. The primary outcome was the incidence of anastomotic leak within 30 days according to the International Study Group of Rectal Cancer (ISGRC) criteria. Secondary outcomes included 30-day readmission, reoperation, length of stay, emergency department visits, and mortality. Outcomes were summarized descriptively.

**Results:**

A total of 287 patients met inclusion criteria, including a heterogeneous population undergoing surgery for both benign and malignant indications. Five patients (1.7%) experienced an anastomotic leak: four Grade B events managed nonoperatively and one event requiring reoperation, with an intact anastomosis noted intraoperatively and classified as Grade C based on reoperation. The 30-day all-cause readmission rate was 3.1%, median postoperative length of stay was 2 days (IQR: 1.25–3), and no deaths occurred. No graft-related adverse events were reported. Exploratory subgroup analyses did not identify meaningful differences in leak rates across operative or clinical characteristics.

**Conclusions:**

In this real-world multicenter pilot study of DHACM applied around the colorectal anastomosis in patients undergoing left-sided colectomy without a protective stoma, low rates of anastomotic leak and 30-day all-cause readmission were observed. Although limited by its retrospective, single-arm design and lack of an internal comparator, these hypothesis-generating findings support the feasibility and tolerability of DHACM as a surgical adjunct at the colorectal anastomosis and provide a rationale for prospective, controlled evaluation in colorectal surgery.

## Introduction

The prevention of anastomotic leak remains one of the most important and persistent challenges in colorectal surgery. Anastomotic failure is associated with substantial clinical and economic consequences, including increased morbidity, mortality, prolonged hospitalization, unplanned reoperations, and long-term functional impairment. In the United States, the overall incidence of anastomotic leaks following colectomy has been estimated to be around 5%–9% (1–4). However, reported rates vary widely, ranging from 0.5% to 30%, depending on factors such as surgical technique, patient comorbidities, anastomotic location, and leak definition (5). Considering the significant clinical and economic burden, strategies to prevent anastomotic leak remain a central focus of colorectal surgical research and practice but few recent technologies have demonstrated consistent, broadly generalizable efficacy in risk reduction.

Left-sided colectomies are performed for a variety of benign and malignant conditions, with diverticulitis and colorectal cancer representing the most common surgical indications (2, 4, 6, 7). Patients with chronic or recurrent diverticulitis often present with dense fibrosis, chronic inflammation, and distorted tissue planes, all of which may compromise tissue perfusion via increased vascular spasm or ongoing mesenteric tension from a foreshortening of the mesentery with chronic inflammation and thus impair anastomotic healing. Additionally, patients with left-sided colorectal cancers may have received neoadjuvant chemotherapy or pelvic radiation, leading to mesenteric devascularization, tissue fibrosis, and impaired microvascular perfusion. Operations for left-sided colorectal cancer may require high vascular ligation, potentially reducing perfusion to the colorectal anastomosis and placing greater reliance on collateral blood flow through the marginal artery. Tumor location may also adversely influence perfusion and increase tension at the anastomotic site. Although the underlying mechanisms differ, both populations share features that predispose to anastomotic ischemia, anastomotic tension and impaired healing. While real-world left-sided colectomy populations are inherently heterogeneous, this clinical reality is not well represented in existing anastomotic leak mitigation studies, which often focus on single-disease cohorts or narrowly defined operative scenarios in attempt to reduce variability.

Numerous adjunctive strategies have been investigated to reduce anastomotic leak risk in colorectal surgery. These include intraoperative perfusion assessment with indocyanine green (ICG) fluorescence angiography, reinforcement of the staple line with bioabsorbable materials, application of tissue sealants, buttressing techniques, and omental flaps. While each may provide incremental benefit in selected contexts, no single method has demonstrated consistent superiority across diverse patient populations (8). Furthermore, despite advances in minimally invasive surgery and enhanced recovery pathways, anastomotic leak rates have not meaningfully declined over the past 15 years (1–4, 6, 7, 9, 10). Diverting stomas are commonly used to mitigate the clinical consequences of a leak, particularly for low pelvic anastomoses; however, diversion does not prevent anastomotic failure, carries its own morbidity, and may lead to permanent stoma in a significant proportion of patients (11–13). Together, these limitations underscore the need for safe, broadly applicable interventions capable of supporting anastomotic healing across heterogeneous left-sided colectomy populations.

Dehydrated human amnion/chorion membrane (DHACM) allografts have been increasingly utilized as surgical adjuncts to support wound healing. Placental tissues are rich in cytokines, growth factors, and tissue inhibitors of metalloproteinases (TIMPs) that are associated with physiological processes relevant to anastomotic healing (14–25). *In vitro* studies have demonstrated DHACM can promote fibroblast proliferation, reduce inflammatory signaling, enhance neovascularization, and modulate stem cell activity (14–23).

Importantly, DHACM does not function as a structural reinforcement or buttress. Instead, it is applied as a biologically derived barrier at the anastomotic site, intended to provide a localized protective microenvironment, and is therefore distinct from buttressing materials, staple-line reinforcements, or sealants. In this context, circumferential application of DHACM may be described as an anastomotic augmentation technique, reflecting its use as a local surgical adjunct at the anastomotic site rather than as a form of structural reinforcement.

Preclinical animal studies further support the potential role of human amniotic membrane in anastomotic healing. Models in rats (26–28), rabbits (29), and dogs (30, 31) have demonstrated benefits in both uncomplicated anastomoses, and in settings complicated by trauma (26), chemotherapy (27), or radiation exposure (29, 31). Across these models, application of human amniotic membrane has been associated with higher anastomotic bursting pressures, decreased inflammation, increased neoangiogenesis and fibroblast activity, improved epithelialization, and enhanced collagenization compared with untreated controls (26–31). Collectively these findings suggest that DHACM may influence the local microenvironment necessary for anastomotic healing, particularly in environments complicated by inflammation, reduced perfusion, or tissue fragility.

Despite this encouraging preclinical evidence, human data evaluating DHACM in colorectal anastomotic healing remain limited. Given this unmet need, a real-world assessment of DHACM use as a protective anastomotic adjunct is warranted. Such an evaluation may help clarify its feasibility, safety, and potential role in supporting anastomotic healing in routine colorectal surgical practice.

The objective of this pilot study was to evaluate postoperative outcomes associated with DHACM application around the colorectal anastomosis in a real-world cohort of patients undergoing left-sided colectomy without a protective stoma.

## Methods

This retrospective, multicenter, observational cohort study evaluated consecutive adult patients who underwent left-sided colectomy with primary colorectal anastomosis and application of dehydrated human amnion/chorion membrane (DHACM) between January 1, 2016, and June 30, 2024. Three board-certified colorectal surgeons practicing at separate institutions contributed cases using a standardized data extraction process.

Eligible patients were initially identified using institutional DHACM tissue utilization logs and subsequently cross-referenced against surgeon-maintained operative case lists and electronic medical records to ensure complete identification of consecutive DHACM-treated cases during the study period. Each participating surgeon provided a complete list of left-sided colectomies performed during the study period, which was reviewed to identify any additional eligible patients who had received DHACM but were not captured in the tissue utilization records. The study was designed as a retrospective descriptive evaluation of postoperative outcomes following DHACM use; therefore, only patients who received DHACM and met the prespecified eligibility criteria were eligible for data abstraction and analysis. Patients were eligible for inclusion if they were 18 years of age or older, underwent a left-sided colectomy (defined as sigmoid colectomy, left colectomy, or low anterior resection) with creation of a primary colorectal anastomosis, received circumferential application of DHACM wrapped around the anastomotic staple line, and had postoperative follow-up data available beyond the index hospitalization sufficient to evaluate 30-day outcomes.

Patients were excluded if they did not undergo a left-sided colectomy, had a history of prior colectomy, received a protective ileostomy or colostomy during the index procedure, lacked postoperative follow-up beyond the index hospitalization, or had insufficient documentation to verify DHACM use. No exclusions were based on surgical indication; therefore, patients undergoing surgery for diverticulitis, colorectal cancer, benign neoplasia, strictures, volvulus, or other left-sided pathology were eligible. Patients in whom DHACM was not applied circumferentially around the anastomosis (e.g., placement adjacent to or interposed near the staple line without full circumferential coverage) were excluded to ensure consistency in application technique.

All procedures were performed by colorectal surgeons certified by the American Board of Colon and Rectal Surgery. The operative approach (laparoscopic, robotic-assisted, or open) was selected based on surgeon preference and patient-specific clinical factors. After standard mobilization and resection, a primary colorectal anastomosis was constructed using a circular stapling device, with stapler selection at the surgeon's discretion. Use of DHACM reflected each surgeon's clinical practice during the study period.

**Figure 1:**
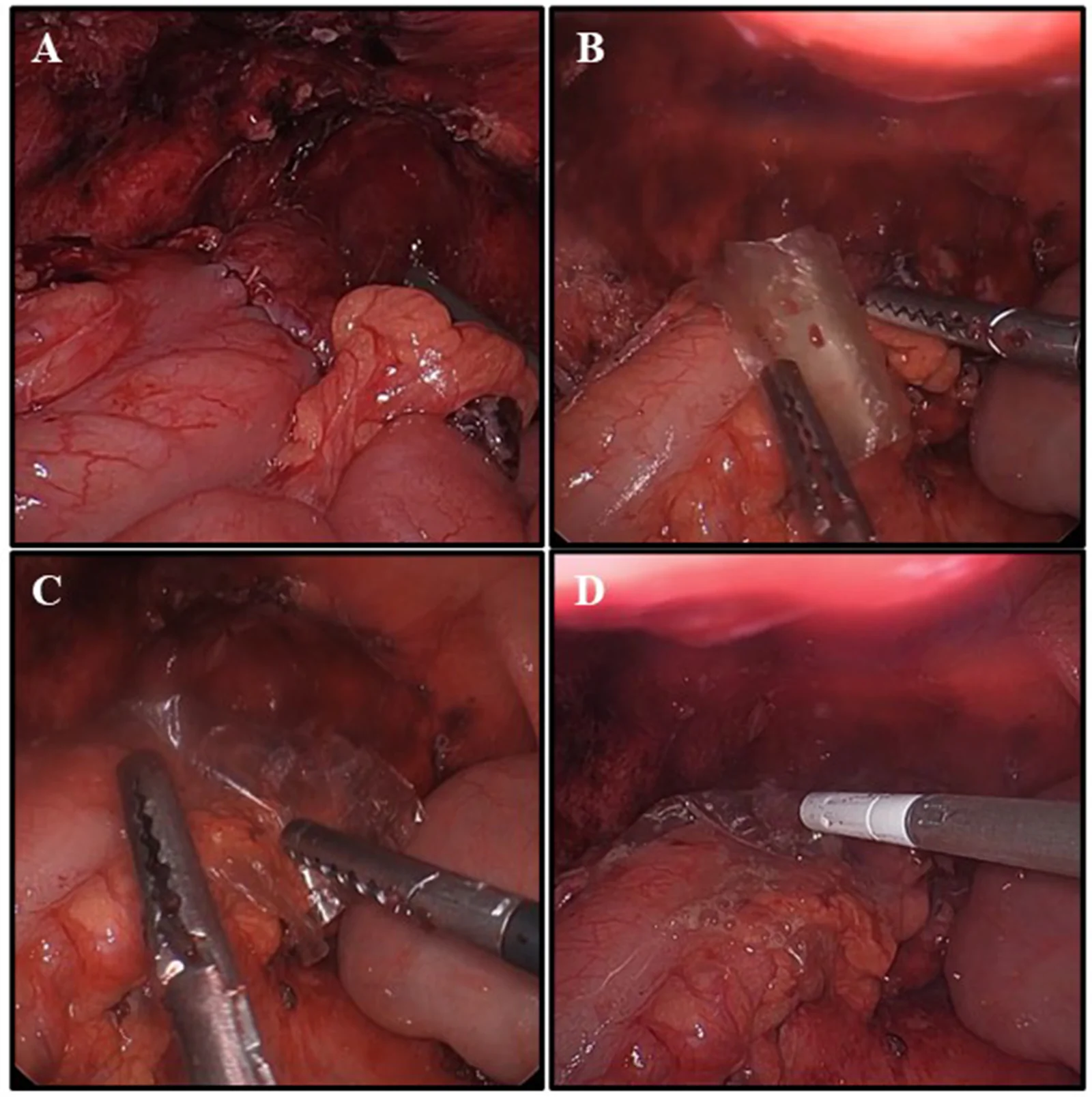
Intraoperative application of dehydrated human amnion/chorion membrane (DHACM) around a colorectal anastomosis during left-sided colectomy. **(A)** Completed colorectal anastomosis following sigmoid colectomy for diverticulitis, demonstrating the circular stapled anastomotic line. (Top Left). **(B)** DHACM introduced into the operative field and positioned adjacent to the completed anastomosis. (Top Right). **(C)** Orientation and circumferential placement of the DHACM to ensure adequate coverage of the anastomotic staple line. (Bottom Left). **(D)** Hydration of the DHACM with saline (or equivalent irrigating solution), demonstrating full coverage of the anastomotic interface. (Bottom Right).

Anastomotic integrity was assessed intraoperatively according to routine surgeon practice under ASCRS Clinical Practice Guidelines and institutional protocols, including pneumatic leak testing and/or ICG fluorescence angiography [used in 98.6% (283/287) of cases]. Following testing, DHACM (AMNIOFIX®, MIMEDX Group, Inc., Marietta, GA, USA), in either a 4 cm × 6 cm or 2 cm × 12 cm configuration, was applied circumferentially around the anastomotic staple line and hydrated per manufacturer instructions (Figure 1). The allograft was used as a surgical adjunct applied at the anastomotic site as a biologically derived barrier and was not intended to alter anastomotic geometry or provide mechanical reinforcement.

When necessary, the graft was trimmed to the required dimensions prior to hydration. Hydration was performed after the graft was positioned *in situ* with the “UP” side facing the surgeon; upon hydration, the material became tacky and typically adhered to the anastomotic line. If additional fixation was required, a single reinforcing stitch and/or tissue adhesives could be used at the surgeon's discretion. Application of the graft generally required less than 3 min. Postoperative care was delivered according to surgeon-directed enhanced recovery pathways based on the patient's clinical course.

The primary outcome was the incidence of an anastomotic leak within 30 days postoperatively. Anastomotic leak was defined in accordance with the International Study Group of Rectal Cancer (ISGRC) as a defect of intestinal wall integrity at the colorectal or colo-anal anastomotic site (including suture or staple lines), resulting in communication between the intra- and extraluminal compartments; a pelvic abscess adjacent to the anastomosis was also considered an anastomotic leak (32).

For purposes of outcome ascertainment in this real-world study, anastomotic leak was operationally identified by one or more of the following within 30 days of surgery: reoperation for suspected leak, reanastomosis, endoscopic stent placement, creation of a colostomy, surgical or percutaneous drainage, and/or radiologic identification of an abscess. This approach reflects real-world clinical management practices and is consistent with commonly applied criteria in benchmark studies (1–4, 6, 7). Leak severity was graded according to ISGRC criteria (Grades A–C) (32). This operational definition was designed to capture clinically managed events and may include cases that do not meet strict ISGRC anatomic criteria for anastomotic disruption; where applicable, such distinctions are explicitly noted in the Results.

Secondary outcomes included 30-day all-cause hospital readmission, emergency department visits within 30 days, reoperation for any cause within 30 days, postoperative length of stay, and 30-day mortality. Exploratory descriptive analyses were performed by surgical indication (e.g., diverticulitis vs. colorectal cancer) and operative characteristics when data were available.

Data were abstracted from operative reports, inpatient and outpatient clinical documentation, and institutional records using de-identified case report forms. To promote data quality and consistency, a random sample comprising more than 30% of study records was re-reviewed by the formal analyst against the original source documentation to verify the accuracy of data abstraction and outcome ascertainment. Each patient was assigned a unique study identification number maintained at the local site, and no protected health information was transferred. Missing data were recorded as “not available” (NA).

The study was conducted in accordance with the Strengthening the Reporting of Observational Studies in Epidemiology (STROBE) guidelines. Prior to data collection, an exemption waiver request was submitted to WCG Institutional Review Board (IRB). On May 1, 2024 WCG IRB approved an exemption request under 45 CFR § 46.104(d)(4), along with a waiver of authorization for use and disclosure of protected health information. Clinical trial number: not applicable.

### Statistical analysis

All consecutive patients who met the inclusion and exclusion criteria were included in the analysis. Because this was a single-arm pilot study designed to estimate the anastomotic leak rate and describe postoperative outcomes in a real-world population, no formal prospective power calculation was performed. Categorical outcomes were summarized as proportions with 95% confidence intervals (CI) using the modified Wald method. Confidence interval calculations were verified using R version 4.5.0 (R Foundation for Statistical Computing, Vienna, Austria). Continuous variables were summarized using means, standard deviations, medians, and ranges as appropriate. In cases of missing data, patients were excluded only from analyses involving the missing variable.

## Results

**Figure 2:**
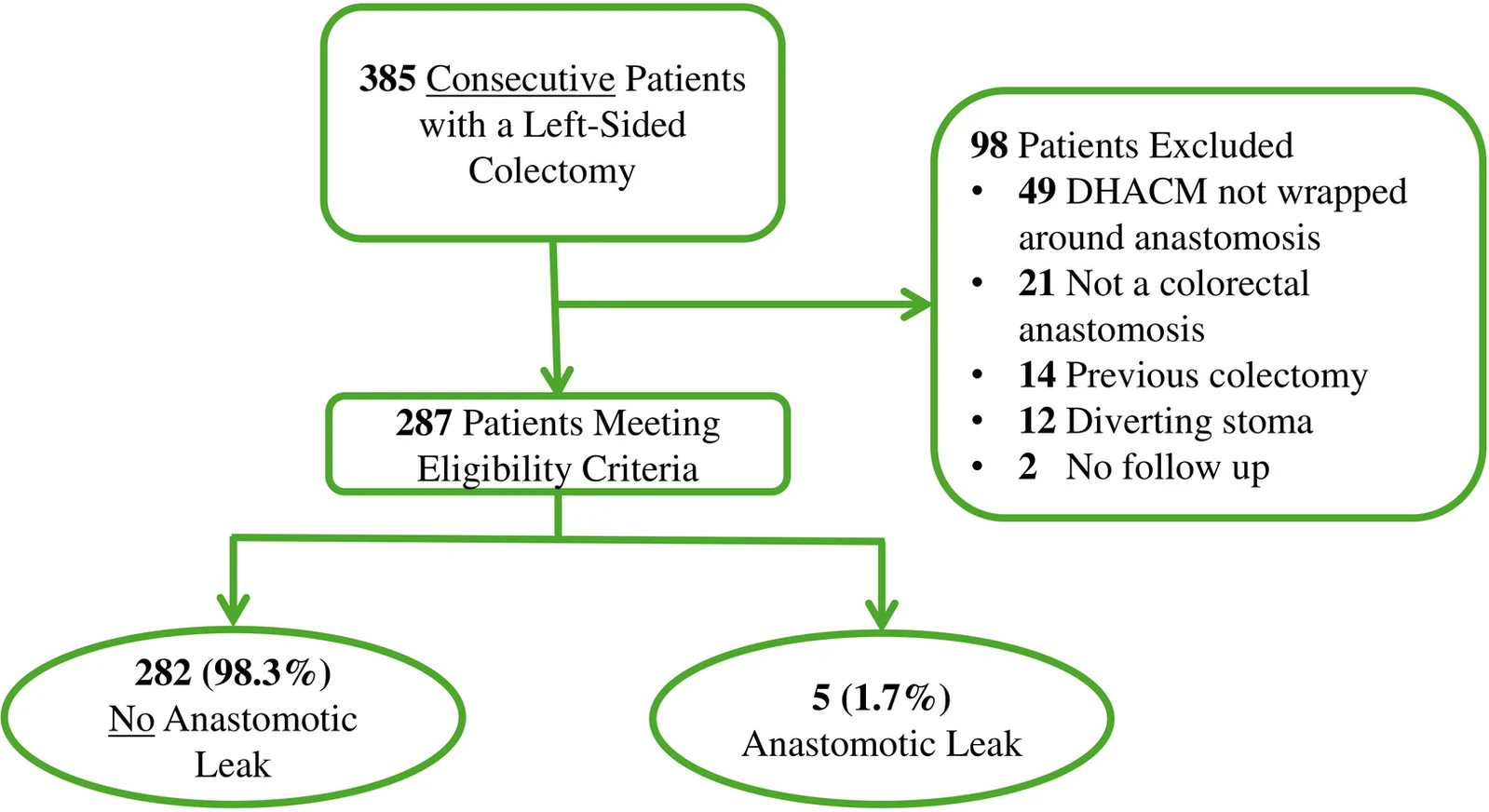
Flow diagram illustrating patient identification, eligibility screening, exclusions, and assembly of the final study cohort of patients undergoing left-sided colectomy with application of dehydrated human amnion/chorion membrane (DHACM).

A total of 385 consecutive patients underwent left-sided colectomy during the study period. Of these, 287 patients met inclusion criteria and were included in the final analysis (Figure 2). In all cases, the colorectal anastomosis was constructed using a circular stapling device (ECHELON™ CIRCULAR Powered Stapler, Ethicon Endo-Surgery, Inc., Cincinnati, OH, USA; ECHELON™ CIRCULAR Manual Stapler, Ethicon Endo-Surgery, Inc., Cincinnati, OH, USA; or Medtronic EEA™ circular stapler with Tri-Staple™, Medtronic, Inc., Mansfield, MA, USA). Eleven minimally invasive cases were converted to open intraoperatively due to factors such as adhesions or difficult pelvic dissection; together with 18 planned open procedures, this resulted in 29 patients (10.1%) whose operations were completed via an open approach. Baseline patient demographics and operative characteristics are summarized in Table 1.

**Table 1:** Patient demographics and baseline clinical characteristics (*n* = 287).

**Patient Demographics (n = 287)**	
Sex, *n* (%): Male Female	151 (52.6%) 136 (47.4%)
Median Age (IQR)	58 (48.5-67) years
Median Body Mass Index (IQR) Number of Patients BMI ≥30 kg/m^2^	29 (25.8-33.9) kg/m^2^ 120 (41.8%)
Comorbidities, *n* (%): Hypertension Respiratory Disease (Asthma, COPD, etc.) Heart Disease (CAD, CHF, etc.) Diabetes Mellitus Non-Colorectal Malignancy (Breast, Lung, Ovarian, etc.) Liver Disease (HBV, HCV, MASH, etc.) Former Smoker Current Smoker	136 (47.4%) 74 (25.8%) 58 (20.2%) 37 (12.9%) 34 (11.8%) 26 (9.1%) 69 (24%) 31 (10.8%)
ASA Physical Status Classification, *n* (%): ASA I ASA II ASA III ASA IV	8 (2.8%) 157 (54.7%) 118 (41.1%) 4 (1.4%)
Colectomy Type, *n* (%): Sigmoid Colectomy Low Anterior Resection	213 (74.2%) 74 (25.8%)
Primary Surgical Indication, *n* (%): Diverticulitis Complicated Diverticulitis Uncomplicated Diverticulitis Acute Diverticulitis Colorectal Cancer Colon Rectum Rectosigmoid Pathologic Stage pT1 pT2 pT3 pT4 Unknown Benign Neoplasia Other Indications	204 (71.1%) 152 (74.5%)* 41 (20.1%)* 11 (5.4%)* 63 (22%) 26 (41.3%)^†^ 19 (30.2%)^†^ 18 (28.6%)^†^ 8 (12.7%)^†^ 7 (11.1%)^†^ 39 (61.9%)^†^ 8 (12.7%)^†^ 1 (1.6%)^†^ 12 (4.2%)8 (2.8%)
Surgical Approach, *n* (%): Laparoscopic Converted to Open Robotic Converted to Open Open	194 (67.6%) 6 (3.1%)^‡^ 75 (26.1%) 5 (6.7%)^‡^ 18 (6.3%)
Circular Stapler Platform, *n* (%): Powered Circular Stapler Manual Circular Stapler (Two-Row) Manual Circular Stapler (Three-Row)	156 (54.4%) 108 (37.6%) 23 (8%)

ASA, American Society of Anesthesiologists; BMI, Body Mass Index; CAD, Coronary Artery Disease; CHF, Congestive Heart Failure; COPD, Chronic Obstructive Pulmonary Disease; HBV, Hepatitis B Virus; HCV, Hepatitis C Virus; IQR, Interquartile Range; MASH, Metabolic Dysfunction-Associated Steatohepatitis.

* Percentage calculated within diverticulitis subgroup.

† Percentage calculated within colorectal cancer subgroup.

‡ Percentage calculated within respective surgical approach.

Among the 287 patients analyzed, five patients (1.7%; 95% CI: 0.6%–4.1%) met the protocol-defined criteria for an anastomotic leak. Four events were classified as ISGRC Grade B leaks, characterized by small intra-abdominal abscesses managed conservatively with antibiotics, with or without percutaneous drainage.

One patient returned to the operating room with pneumoperitoneum and a pelvic hematoma and was classified as Grade C based on the need for reoperation. Intraoperative assessment demonstrated an intact anastomosis without evidence of disruption or fecal contamination. This event met the study's predefined criteria for anastomotic leak based on reoperation for suspected leak but may not meet strict ISGRC definitional criteria. In a sensitivity analysis excluding this event, the anastomotic leak rate was 4/287 (1.4%; 95% CI: 0.4%–3.6%). A descriptive summary of patients experiencing anastomotic leaks is provided in Table 2.

**Table 2:** Characterization of anastomotic leak events Per ISGRC criteria (32) (*n* = 5).

Patient	Leak grade (32)	Presentation & timing	Management	Key comorbidities	Surgical characteristics
1	Grade B (abscess)	Female, 48 years; readmitted POD 24 with intra-abdominal abscess concerning for anastomotic leak on CT	Antibiotics only; no drainage or reoperation	Hypertension	Sigmoid colectomy for diverticulitis; ASA II; robotic approach; powered circular stapler
2	Grade B (abscess)	Male, 49 years; readmitted POD 23 with abdominal pain and small contained fluid collection on CT	Antibiotics only; no drainage or reoperation	Diabetes (HbA1c 6.7), hypertension, cardiovascular disease; former smoker	Sigmoid colectomy for diverticulitis; ASA III; laparoscopic approach; powered circular stapler
3	Grade B (abscess)	Female, 71 years; readmitted POD 7 with intra-abdominal abscess on CT	Percutaneous drainage and antibiotics; no reoperation	Hypertension, asthma	Sigmoid colectomy for diverticulitis; ASA III; laparoscopic approach; powered circular stapler
4	Grade B (abscess)	Female, 76 years; readmitted POD 9 for acute respiratory failure; incidental perianastomotic fluid collection identified on CT	Antibiotics only; no drainage or reoperation	Diabetes (HbA1c 7.6), hypertension	Low anterior resection for tubular adenoma; ASA II; open approach; manual circular stapler
5	Grade C* (reoperation)	Male, 18 years; returned to operating room POD 5 with pneumoperitoneum and pelvic hematoma	DLI; Anastomosis intact; no fecal contamination	Hirschsprung's disease	Sigmoid colectomy for volvulus; ASA II; open approach; manual circular stapler

ASA, American Society of Anesthesiologists; CT, Computed Tomography; DLI, Diverting Loop Ileostomy; POD, Post-Operative Day.

* Classified as Grade C based on reoperation; intraoperative findings did not demonstrate a clear anastomotic defect and may not meet strict ISGRC definitional criteria.

**Figure 3:**
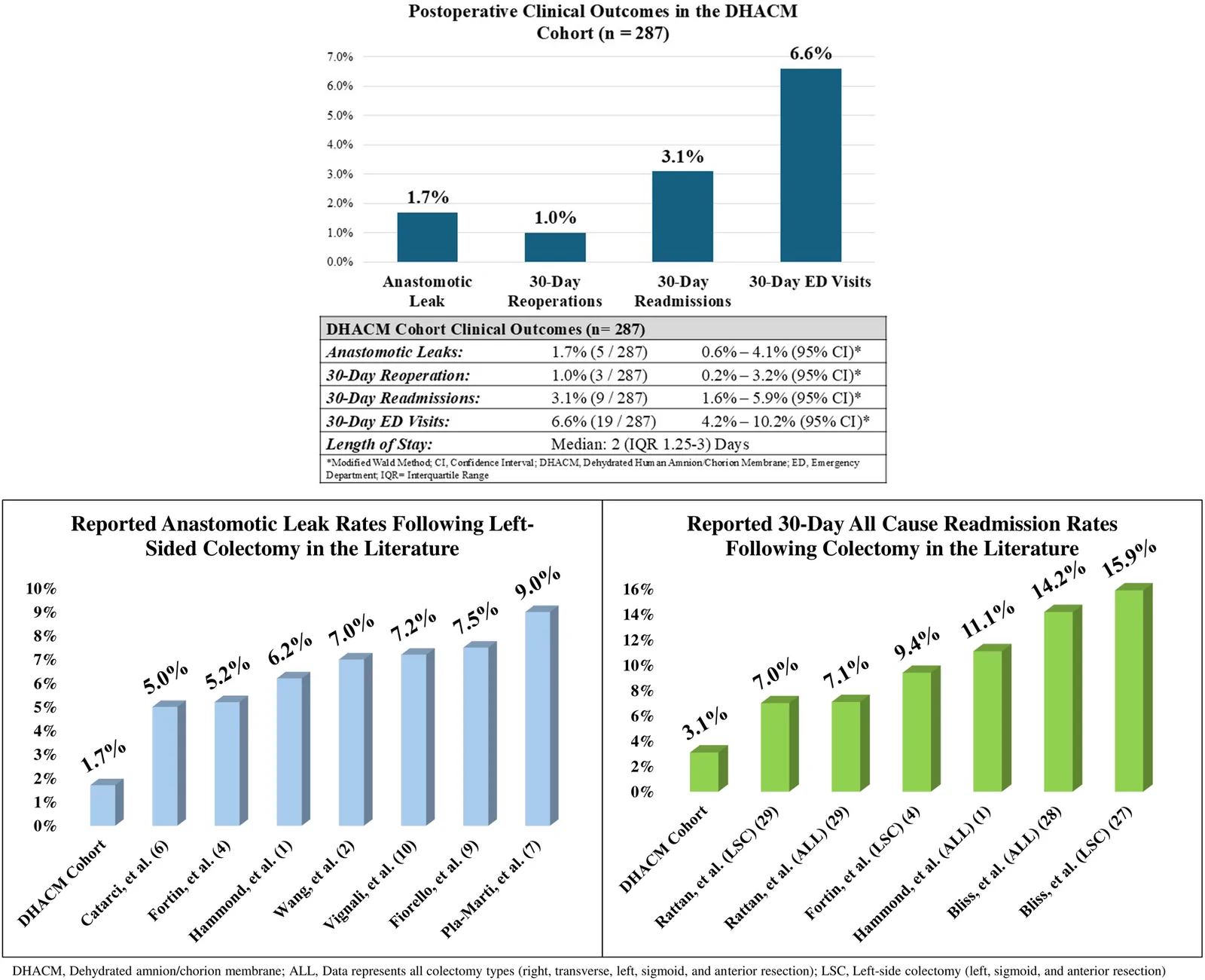
Observed 30-day postoperative outcomes in patients undergoing left-sided colectomy with circumferential application of dehydrated human amnion/chorion membrane (DHACM). Outcomes are presented as percentages of the study cohort (*n* = 287).

During the 30-day postoperative period, 19 patients (6.6%) presented to the emergency department, with 15 visits considered potentially related to the index operation. The 30-day all-cause hospital readmission rate was 3.1% (9/287), and 1.0% (3/287) of patients required reoperation within 30 days. The median postoperative length of stay was 2 days (IQR: 1.25–3). No deaths occurred in the cohort and no graft-related adverse events were reported. A summary of key postoperative outcomes is presented in Figure 3.

Exploratory analyses showed comparable leak rates across most patient and operative subgroups (Table 3). Numerically higher rates were observed in several small subgroups, including patients undergoing open surgery and those with benign neoplasia or other surgical indications; however, these findings were based on one or two events and should be interpreted with considerable caution. Accordingly, subgroup results should be viewed as descriptive.

**Table 3:** Exploratory subgroup analysis of anastomotic leak events (*n* = 5).

Patient subset	Anastomotic leak rate
Colectomy type (n, %):	
Sigmoid colectomy	1.9% (4/213)
Low anterior resection	1.4% (1/74)
Primary surgical indication (*n*, %):	
Diverticulitis	1.5% (3/204)
Cancer	0% (0/63)
Benign neoplasia	8.3% (1/12)
Other indications	12.5% (1/8)
Surgical approach (*n*, %):	
Laparoscopic	1.0% (2/194)
Robotic	1.3% (1/75)
Open	11.1% (2/18)
Circular stapler platform (*n*, %):	
Powered circular stapler	1.9% (3/156)
Manual circular stapler	1.5% (2/131)

Subgroup analyses were exploratory and descriptive. Formal statistical comparisons were not performed due to the small number of anastomotic leak events.

## Discussion

Anastomotic leak remains one of the most serious complications following colorectal surgery. Although uncommon in most surgical series, its downstream clinical and economic consequences are disproportionate, including increased postoperative morbidity and mortality, prolonged hospitalization, unplanned reoperations, readmissions, and long-term impairment in quality of life (1, 3, 4). Even clinically “contained” leaks frequently lead to a cascade of interventions, such as repeat imaging, percutaneous drainage, prolonged antibiotic therapy, delayed recovery, and hospital readmission. Furthermore, clinically obvious leaks often require reoperation, permanent stoma formation, and significant morbidity to a patient's quality of life. Despite advances in minimally invasive techniques, intraoperative perfusion assessment, and enhanced recovery pathways, analyses from large administrative datasets suggest that reported anastomotic leak rates have remained largely unchanged since 2008 (1, 2, 4).

The present pilot study evaluated real-world postoperative outcomes observed following application of dehydrated human amnion/chorion membrane (DHACM) around the colorectal anastomosis following left-sided colectomy. The cohort included a heterogeneous patient population undergoing surgery for both benign and malignant indications, reflecting contemporary clinical practice. To our knowledge, this study represents one of the few published multicenter real-world evaluations of DHACM use in left-sided colorectal surgery.

Key limitations of this study are its observational design and the absence of an active comparator group. Reported anastomotic leak rates following left-sided colectomy generally range from approximately 5%–9% across contemporary studies, with variability attributable to differences in patient populations, operative techniques, study design, outcome definitions, methods of outcome ascertainment, and perioperative management (1–4, 6, 7, 9, 10). In the present study, the observed anastomotic leak rate was 1.7%. Given the single-arm, retrospective design and absence of a contemporaneous control group, this finding should be interpreted descriptively, and cross-study comparisons should be made with caution.

Reported 30-day readmission rates following left-sided colectomy similarly vary across studies, with published estimates generally ranging from approximately 7%–15.9%. These differences reflect variation in patient characteristics, perioperative care pathways, study design, and methods of follow-up and outcome ascertainment (1, 4, 33, 34). The 30-day readmission rate observed in this cohort was 3.1% and should be interpreted within the same context. As with anastomotic leak, comparisons to published rates are descriptive and should not be interpreted as evidence of comparative effectiveness.

The median postoperative length of stay observed in this cohort was 2 days, compared with approximately 5.3 days reported in Premier Healthcare Database analyses from the same time period (4). Whether the observed readmission and length-of-stay outcomes primarily reflect the low incidence of anastomotic leak or other unmeasured clinical or institutional factors cannot be determined from this study and warrants further investigation in controlled or comparative study designs.

DHACM is used as a surgical adjunct rather than a mechanical reinforcement and is therefore distinct from buttressing materials, staple-line reinforcements, or sealants. Its proposed utility relates to the preservation and elution of biological components within the allograft. *In vitro* studies have demonstrated that DHACM contains and releases a broad array of growth factors, including vascular endothelial growth factor, basic fibroblast growth factor, platelet-derived growth factor, and transforming growth factor-*β*, which have been associated with angiogenesis, fibroblast recruitment, and collagen synthesis (14–23). These biological activities have been associated with increased anastomotic strength, enhanced collagen deposition, and improved histologic healing in experimental models (24, 25, 35–38). Fibroblasts, which play a central role during the proliferative phase of anastomotic wound healing, are attracted by growth factors present in DHACM and contribute to extracellular matrix deposition and tissue remodeling (14–24, 36, 39).

Preclinical studies further suggest placental allografts may exert protective effects on anastomotic healing under adverse conditions, including trauma, radiation exposure, and cytotoxic injury (26, 27, 29, 31). Experimental animal models have demonstrated associations between placental allograft application and reduced inflammatory infiltration, increased neovascularization, enhanced fibroblast activity, and increased collagen deposition (26–31). These effects have been attributed, in part, to cytokines, tissue inhibitors of metalloproteinases (TIMPs), and growth factors preserved within placental tissue (14–25). Modulation of matrix metalloproteinase activity early in the healing process has been associated with increased anastomotic bursting pressure in experimental models (24, 25, 37).

Collectively, these biological observations provide a plausible mechanistic rationale for the use of DHACM in surgical settings, although a direct causal contribution to the outcomes observed in this cohort cannot be established from this study design. The precise contribution of DHACM to anastomotic healing in humans remains difficult to isolate given the multifactorial nature of surgical healing and the influence of variables such as perfusion, anastomotic tension, operative technique, and patient-specific comorbidities. Accordingly, any mechanistic interpretation should be considered exploratory rather than confirmatory.

The strengths of this study include the inclusion of consecutive patients to reduce selection bias; a large, heterogeneous real-world cohort reflective of contemporary practice; standardized data abstraction across multiple surgeons at diverse institutions; and exclusive inclusion of patients undergoing left-sided colectomy without diversion, allowing evaluation of anastomotic healing without the confounding effect of protective stomas.

Several limitations warrant consideration. As a retrospective single-arm study, causal inference cannot be established, and comparisons to historical benchmarks are descriptive rather than statistical. In addition, because the study population was restricted to patients who received DHACM, a contemporaneous non-DHACM cohort was not collected, precluding internal comparisons between treated and untreated patients. Retrospective data collection may have resulted in incomplete documentation of certain confounders, including prior chemotherapy or radiation and anastomotic height, precluding reliable evaluation of these variables. The low number of observed anastomotic leaks limits the ability to detect subgroup differences or identify predictors of leak. Operative technique, including use of fluorescence angiography, stapler platform, and adjunctive measures, varied across surgeons, reflecting real-world practice while introducing clinical heterogeneity. Although more than 30% of records underwent source verification, data abstraction and outcome adjudication were not performed independently or in a blinded manner. Finally, unmeasured confounding cannot be excluded. The favorable outcomes observed in this cohort may reflect multiple factors, including patient selection, institutional practice patterns, perioperative management, outcome ascertainment, or other unmeasured variables in addition to the use of DHACM. Given the observational single-arm design, the relative contribution of these factors cannot be determined.

Although this study was not prospectively powered to test a formal hypothesis, a *post hoc* sample size assessment was performed to provide context regarding the size of the study population. Using an illustrative historical anastomotic leak rate of approximately 7% derived from contemporary literature and a hypothetical two-thirds relative reduction in leak rate (7.0%–2.3%), approximately 242 patients would be required to achieve 90% power using a one-sample test of proportions (two-sided *α* = 0.05) (2). This calculation was performed after completion of the study, was not used to determine the sample size, and is presented solely to provide context for future study design.

Given the heterogeneous patient population and absence of an internal comparator, formal risk-adjusted or sensitivity analyses were not performed; with only five observed anastomotic leak events, such models would be severely underpowered and statistically unreliable. These methodological constraints further reinforce the characterization of this work as hypothesis-generating pilot data, intended to inform the design of adequately powered prospective trials rather than to establish comparative efficacy.

Taken together, these findings describe outcomes in patients undergoing left-sided colectomy without a protective stoma with use of DHACM at the colorectal anastomosis. These observations support the feasibility of this approach and provide a rationale for further prospective evaluation using controlled and comparative study designs.

## Conclusion

In this real-world pilot study of DHACM use in patients undergoing left-sided colectomy without a protective stoma, low rates of anastomotic leak and 30-day all-cause readmission were observed across both benign and malignant indications. Although limited by its single-arm, retrospective design, these findings support the feasibility and tolerability of DHACM as a surgical adjunct at the colorectal anastomosis and are hypothesis-generating. Prospective controlled studies are warranted to further evaluate outcomes and better define its potential role in colorectal surgery.

## Data Availability

The raw data supporting the conclusions of this article will be made available upon reasonable request, without undue reservation, subject to applicable institutional and ethical requirements.
